# Antimicrobial Susceptibility Profiles of Commensal *Staphylococcus* spp. Isolates from Turkeys in Hungarian Poultry Farms Between 2022 and 2023

**DOI:** 10.3390/antibiotics14020200

**Published:** 2025-02-14

**Authors:** László Kovács, Ábel Szabó, Franciska Barnácz, Bence Csirmaz, Ákos Jerzsele, Ádám Kerek

**Affiliations:** 1 Department of Animal Hygiene, Herd Health and Mobile Clinic, University of Veterinary Medicine, István utca 2, H-1078 Budapest, Hungary; kovacs.laszlo@univet.hu; 2 Poultry-Care Kft., H-5052 Újszász, Hungary; 3 National Laboratory of Infectious Animal Diseases, Antimicrobial Resistance, Veterinary Public Health and Food Chain Safety, University of Veterinary Medicine, István utca 2, H-1078 Budapest, Hungary; jerzsele.akos@univet.hu; 4 Department of Pharmacology and Toxicology, University of Veterinary Medicine, István utca 2, H-1078 Budapest, Hungary; szabo.abel@student.univet.hu (Á.S.); barnacz.franciska@student.univet.hu (F.B.); csirmaz.bence@student.univet.hu (B.C.)

**Keywords:** *Staphylococcus*, antimicrobial resistance, minimum inhibitory concentration, MIC, poultry, turkeys, Hungary

## Abstract

**Background:** The poultry industry is one of the most rapidly growing sectors, producing the highest amount of animal-derived protein per unit time while also being the second-largest consumer of antibiotics. The widespread and accelerating spread of antimicrobial resistance (AMR) underscores the necessity of regular monitoring studies. Periodic assessments, especially focusing on commensal strains, can serve as indicators of emerging resistance patterns. **Methods:** This study assesses the antimicrobial susceptibility profiles of putative commensal *Staphylococcus* strains (*n* = 166) isolated from large-scale turkey flocks in Hungary using minimal inhibitory concentration (MIC) determination. The isolated strains were tested against antibiotics of veterinary and public health importance. The results were analyzed using the Kruskal–Wallis test and the Mann–Whitney *U* test, as well as *t*-tests. Additionally, correlation analysis and principal component analysis were performed. **Results:** Our findings revealed the highest resistance rates to tiamulin (90.4%), doxycycline (79.5%), and enrofloxacin (68.7%). **Conclusions:** These results reflect the extensive antibiotic use in the poultry sector, which contributes to the widespread presence of antimicrobial resistance. As regular monitoring and the identification of trends can aid in mitigating the spread of resistance, these findings should be complemented by data on antibiotic usage at the surveyed farms in further studies. The observed resistance rate of 18.1% to vancomycin is particularly concerning from a public health perspective, given that comparative human data show only a 0.05% resistance rate. Additionally, for multidrug-resistant strains, next-generation sequencing should be utilized to elucidate the genetic mechanisms underlying resistance, particularly in strains exhibiting high levels of resistance to vancomycin, which is of critical importance in human medicine, as well as to the critically important enrofloxacin and the widely used doxycycline and tiamulin. However, the limitations of the study should also be acknowledged, including the relatively small sample size, which is significantly lower than that of available human data, as well as the spatial distribution of the samples.

## 1. Introduction

Antimicrobial resistance (AMR) in livestock has become a global concern, with pharmaceutical companies and drug developers unable to keep pace with the increasing prevalence of resistant strains. In the United States alone, the annual costs of combating resistant bacterial infections are estimated at USD 21–34 billion per year [1]. However, predictions indicate that, relatively speaking, Africa will bear the most significant economic burden, while the world’s economic output (GDP) is projected to decline by USD 1 trillion by 2050 [2].

In the European Union, AMR of bacteria in food-producing animals and bacteria isolated from meat surfaces is governed by Regulation (EC) No 178/2002 [3], complemented by Directive 2003/99/EC, which addresses zoonoses and their surveillance. These regulations emphasize the importance of collecting comparable data on zoonotic and commensal bacteria [4]. The monitoring and reporting of AMR in zoonotic and commensal bacteria are further governed by the 2013/652/EU Implementing Decision, which aligned with the European Commission’s 2011–2016 action plan and extended through 2020. This initiative prioritizes AMR surveillance from a public health perspective [5].

Bacteria can develop resistance through various mechanisms, including chemical inactivation of antimicrobials, efflux pump systems, reduced uptake, or target site modification that negates drug efficacy [6]. The rapid evolution of these mechanisms cannot be attributed solely to spontaneous mutations; rather, horizontal gene transfer has been noted to play a critical role [7]. Horizontal gene transfer involves the exchange of genetic material between unrelated organisms, often mediated by mobile genetic elements [8] such as plasmids and bacteriophages [9].

Multiple factors contribute to the rapid spread of resistance, which can be categorized by the two main causes: the quantity of antimicrobials used and the appropriateness of their application [9]. Studies in humans have shown an inverse correlation between AMR prevalence and socioeconomic indicators such as better-quality infrastructure, higher GDP, and increased public health expenditure [10]. Conversely, poor governance and higher climatic temperatures correlate positively with increased resistance rates. As a consequence, it has been argued that merely reducing antibiotic consumption is insufficient to curb AMR; effective infection control measures to halt the spread of resistant strains and resistance genes are even more critical [10].

As the swine [11] and poultry industries are among the highest consumers of antibiotics, these sectors should implement strategies emphasizing reduction and replacement [12]. Given the challenges posed by AMR, coupled with consumer demand for sustainable practices, the poultry industry must prioritize the search for viable antibiotic alternatives [13]. Research into plant-derived supplements, such as essential oils [14] or plant extracts [15,16], has shown promise in reducing the emergence of resistant strains. Some studies have also highlighted that medium-chain fatty acids damage bacteria [17]. Similarly, antimicrobial peptides [18] and natural compounds like propolis have demonstrated varying degrees of efficacy [19,20,21]. Equally important, especially for large-scale livestock operations, is the implementation of robust biosecurity measures [22,23], as wild birds have been identified as reservoirs for antimicrobial resistance genes [24]. Proper pharmacokinetic and pharmacodynamic studies are crucial for guiding effective therapy and preserving the long-term efficacy of antimicrobials [25].

*Staphylococcus* species are Gram-positive bacteria with a broad host spectrum, colonizing the skin, mucosa, and respiratory tracts of humans and birds [26,27]. Although primarily considered commensal organisms, their pathogenicity can increase when the host’s defenses are compromised [28]. Among these, *Staphylococcus aureus* (*S. aureus*) is the most invasive, capable of causing a wide range of diseases, from skin infections to severe conditions, including toxic shock syndrome and sepsis [29,30,31]. Under antibiotic pressure, these strains can rapidly evolve into methicillin-resistant *S. aureus* (MRSA), with resistance conferred by the *mecA* gene and its homolog *mecC*, often carried on mobile genetic elements [32,33]. These strains pose significant risks not only to public health, through hospital- and community-acquired infections, but also to livestock-associated (LA) MRSA strains, which have greater zoonotic potential and which are becoming a global issue [34,35,36]. It is also important to note that, in addition to MRSA, the most common zoonotic species within the genus include *Staphylococcus pseudintermedius* [37] and its methicillin-resistant form *Staphylococcus pseudintermedius* [38], as well as *Staphylococcus intermedius* [39].

In poultry products, the primary concern is the potential spread of resistant strains to other animals or humans [40], and the secondary concern is the production of enterotoxins, which can cause foodborne illnesses in humans. Improper preparation of raw poultry meat can lead to cross-contamination, and the consumption of undercooked meat also increases the risk of MRSA infections [41,42]. Recent studies have identified high AMR carriage rates in both commercial poultry and backyard flocks, raising concerns about their potential as reservoirs for human infections [43]. The AMR genes carried on mobile genetic elements constitute approximately 25% of the *S. aureus* genome, encoding numerous virulence factors and resistance mechanisms [44]. These mobile genetic elements (MGEs) play a crucial role in the adaptability and survival of bacteria [45]. An American study reported that 96% of *S. aureus* strains isolated from poultry were resistant to at least one antibiotic [46].

Turkeys are an important food-producing species, raised both for their meat and as breeding stock for hatching egg production. Turkey meat is the fourth-most commonly consumed meat in Canada and ranks second globally [47]. Several outbreaks linked to turkey meat consumption in Canada and the USA have been associated with *Salmonella enterica* serovars, including Reading [48,49], Schwarzengrund [50], Heidelberg [51,52], and Hadar [53]. Multidrug-resistant (MDR) *Salmonella enterica* serotype Reading outbreaks associated with turkey meat occurred between 2017 and 2020 in Canada and between 2017 and 2019 in the USA [48,49,54]. Canada has implemented an integrated antimicrobial resistance surveillance system (CIPARS) which monitors resistance profiles in turkey-derived *Campylobacter* spp., *Escherichia coli*, and *Salmonella enterica* isolates. This system has documented strains resistant to at least one antimicrobial group [55], including critically important antibiotics (fluoroquinolones, third- and fourth-generation cephalosporins, colistin) as identified by the World Health Organization (WHO) and Health Canada [56,57]. CIPARS also reported differences in AMR profiles between broiler chickens and turkeys within the poultry sector [55].

Although *S. aureus* is an opportunistic pathogen, it can also persist in a commensal state under certain conditions. In this study, we isolated *S. aureus* strains from the tracheal mucosa of clinically healthy poultry. Since these animals did not show any clinical signs of disease, we refer to these strains as putative commensals. However, further investigations would be needed to fully confirm their commensal nature. Given the undeniable economic importance and public health significance of turkeys, our study aims to assess the AMR profiles of *Staphylococcus* strains isolated from large-scale turkey farms in Hungary. Additionally, we compared these findings with human resistance data, fostering integrated understanding between veterinary and public health sectors within the One Health framework.

## 2. Results

### 2.1. Regional Distribution and Origin of Samples Received

In this study, a total of 166 putative commensal *Staphylococcus* strains were isolated from 21 large-scale turkey farms in Hungary. Of these farms, seven (33.3%) were breeding facilities, while 14 (66.7%) were meat-producing broiler turkey farms. The majority of samples originated from the Dél-Alföld region (26.5%), followed by the Észak-Alföld region (24.7%). Respiratory samples accounted for 96.9% of the isolates, with only 3.1% obtained from cloacal swabs. Most isolates (81.9%) were derived from meat-producing flocks, while 18.1% were from breeding flocks, reflecting a similar distribution across age groups. Additionally, 86.1% of the samples came from small-sized farms (5001–50,000 birds), while 13.9% were collected from medium-sized farms (50,001–100,000 birds).

### 2.2. Antimicrobial Susceptibility Testing

**Figure 1:**
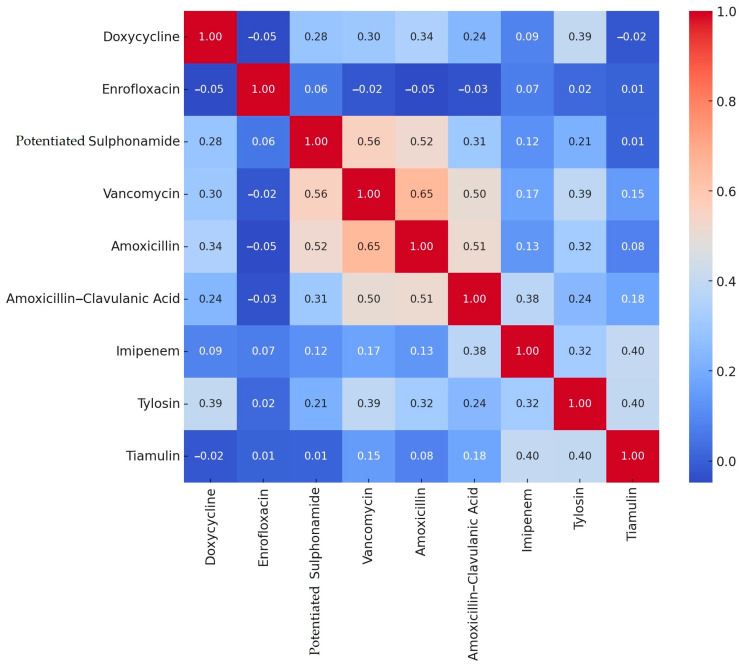
Results of correlation analysis of antimicrobial resistance of *Staphylococcus* strains (*n* = 166) received and isolated from turkey colonies by drug and plotted on heat map. The correlation analysis reveals the strength of the relationships between resistance to each antimicrobial. A deeper red color indicates a stronger positive correlation, meaning that the emergence of resistance to one drug is likely associated with the emergence of resistance to another. The strongest positive correlations were observed between amoxicillin and vancomycin, amoxicillin–clavulanic acid and vancomycin, vancomycin and potentiated sulphonamide, amoxicillin and potentiated sulphonamide, as well as amoxicillin and amoxicillin–clavulanic acid.

Following the determination of the minimum inhibitory concentration (MIC) values, resistance levels were assessed for antibiotics with established clinical breakpoints, and correlation analyses were performed (Figure 1). In the present study, a strong positive correlation was observed between amoxicillin and vancomycin (0.65), as well as between amoxicillin and potentiated sulfonamide (0.52) and between amoxicillin and amoxicillin–clavulanic acid (0.51). No significant negative correlations were observed; the strongest negative correlations, though negligible in magnitude, were between amoxicillin and enrofloxacin (−0.05) and between enrofloxacin and doxycycline (−0.05).

**Figure 2:**
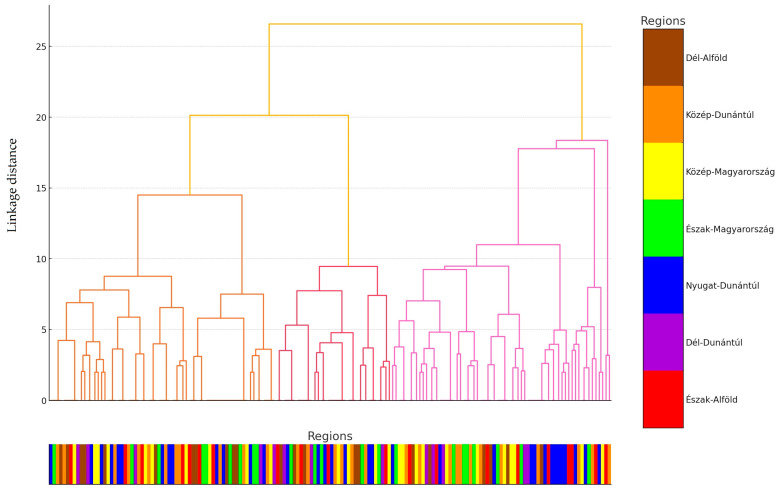
The dendrogram represents the cluster analysis of *Staphylococcus* strains (*n* = 166) isolated from turkey flocks. To enhance clarity, given the large sample size, each sample was categorized based on its region of origin. Regions were color-coded, and the corresponding region of each sample is indicated below the horizontal axis using the assigned color code.

Subsequently, hierarchical cluster analysis was performed, and the results were visualized using a dendrogram (Figure 2).

**Figure 3:**
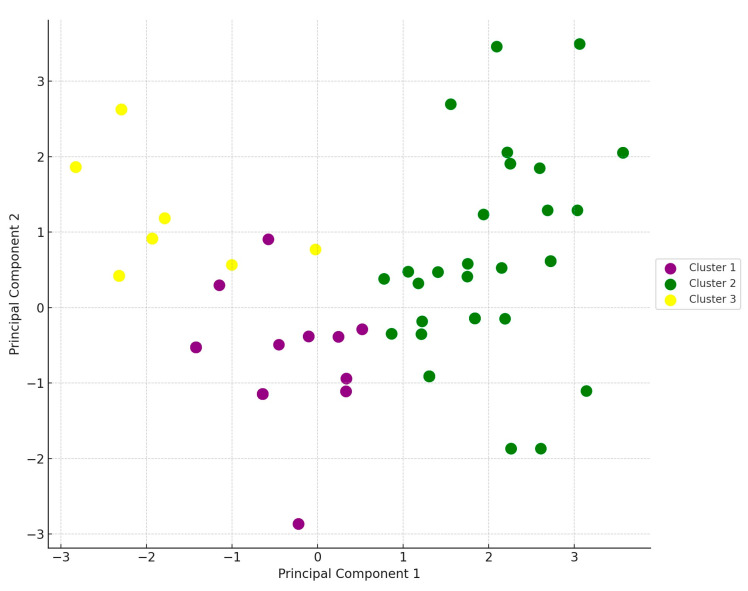
Visual representation of sample distribution across the three main clusters for *Staphylococcus* strains (*n* = 166) isolated from turkey flocks.

Following the cluster analysis, we performed principal component analysis (PCA), a statistical method used to reduce data dimensionality and uncover relationships among variables (Figure 3). As a result, the samples were categorized into three main clusters.

We performed a two-sample *t*-test for each grouping criterion to assess the level of resistance by active antibiotics and determine whether significant differences exist between groups (Table 1). The most notable factors were utilization type (broiler vs. breeding) and the associated age groups (juvenile vs. adult). In these cases, significant differences were observed for all active substances except enrofloxacin and potentiated sulfonamides. Similarly, flock size was a determining factor, with comparisons between small (5001–50,000) and medium (50,001–100,000) farms revealing notable differences. In contrast, the sample source (respiratory vs. cloacal) had the least impact, with significant differences observed only for doxycycline (0.0067) and potentiated sulfonamides (0.0097).

From the MIC values obtained during the determination process, a frequency table was created (Table 2). This table includes clinical breakpoints, MIC_50_ and MIC_90_ values, and epidemiological cut-off value (ECOFF) values as defined by European Committee on Antimicrobial Susceptibility Testing (EUCAST).

The MIC_50_ and MIC_90_ values are used to measure the efficacy of antibiotics. These values are statistical measures of MIC that indicate the concentration of an agent required to inhibit the growth of a certain percentage of the microbial population in the tested sample. The MIC_50_ is the minimum concentration that inhibits the growth of 50% of the tested microbial population, while the MIC_90_ represents the minimum concentration required to inhibit 90% of the population. These metrics provide critical insights into the distribution of susceptibility within the microbial population. In our findings, the MIC_50_ values for amoxicillin–clavulanic acid, imipenem, tilozin, and vancomycin remained below the clinical breakpoints, indicating their potential efficacy against the tested strains.

We compared the values obtained in our study with the ECOFF values defined by EUCAST. ECOFF is a critical metric in antimicrobial resistance research. These values help determine the natural distribution of susceptibility to a particular antimicrobial agent within a microbial population, identifying isolates that are naturally sensitive or resistant. ECOFF represents the MIC value that separates wild-type microorganisms from those that possess resistance mechanisms to a specific antimicrobial agent. Wild-type isolates are those that do not have acquired or mutation-driven resistance mechanisms. In our study, the MIC_50_ values for amoxicillin-clavulanic acid, imipenem, and vancomycin were below their respective ECOFF values, indicating that the majority of isolates tested for these agents may still be within the wild-type population.

The frequency of MIC values for agents without clinical breakpoints is summarized in Supplementary Table S1.

**Figure 4:**
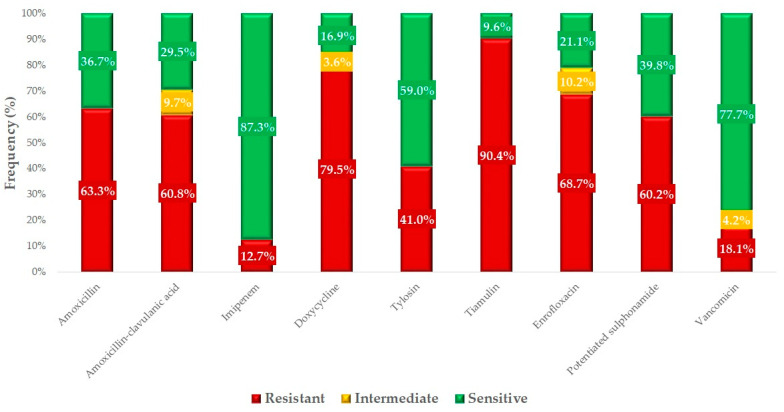
Antimicrobial resistance profile of *Staphylococcus* isolates (*n* = 166) from turkey farms.

We determined the resistance rates for each antimicrobial agent with defined clinical breakpoints (Figure 4). The highest resistance was observed for tiamulin (90.4%), followed by doxycycline (79.5%). Resistance levels for amoxicillin (63.3%) and amoxicillin–clavulanic acid (60.8%) were nearly identical, suggesting that a significant proportion of the isolates are not β-lactamase producers. However, the proportions of isolates resistant to imipenem (12.7%) and vancomycin (18.1%) are particularly concerning, because of the public health significance of these antibiotics.

**Figure 5:**
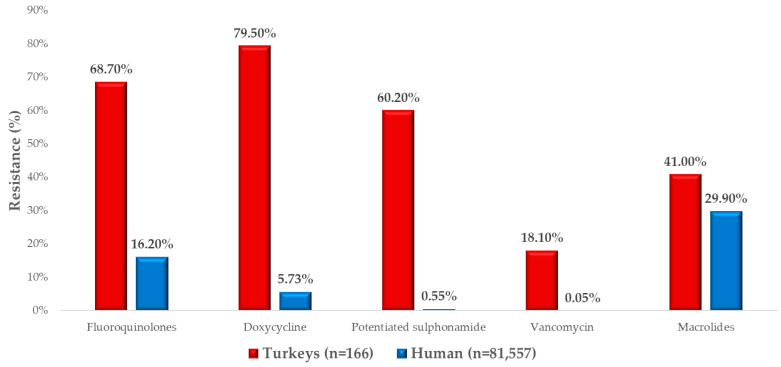
Comparison of resistance of turkey-isolated strains and available human resistance data.

We compared our results with human AMR data from National Public Health and Pharmaceutical Center (Figure 5). The most comparable resistance rate was observed for macrolides, with turkey isolates showing a resistance rate of 41% and human isolates showing a rate of 29.9%. However, significant differences in resistance were noticeable for the other four antimicrobials we tested, with AMR in the samples taken from turkeys being alarmingly higher than in those collected from humans.

Resistance to fluoroquinolones was 68.7% in turkeys, compared to a much lower resistance rate of 16.2% in humans. Even more dramatic differences were observed for resistance to doxycycline: 79.5% in turkey isolates but only 5.73% in human isolates. Similarly, potentiated sulfonamides showed a resistance rate of 60.2% in turkey samples, compared to just 0.55% in humans. Although lower, the 18.1% vancomycin resistance observed in turkey isolates is alarming, as only 0.05% resistance was detected in human healthcare settings.

## 3. Discussion

Our findings suggest that *S. aureus* can be present in the tracheal mucosa of healthy poultry without causing disease. However, as *S. aureus* is a well-known opportunistic pathogen, these isolates cannot be definitively classified as purely commensal without further virulence factor analysis and in vivo pathogenicity testing. Our study focused on the antimicrobial susceptibility testing of putative commensal *Staphylococcus* strains (*n* = 166) isolated from turkeys. A limitation of this study is that it focuses exclusively on *Staphylococcus* species. However, it is important to acknowledge that interspecies communication can facilitate the spread of antibiotic resistance, meaning that analyzing a single species in isolation may introduce biases.

While amoxicillin is widely used in the poultry industry, ampicillin is more commonly applied in human healthcare and better represents the resistance profile in that sector. Nevertheless, the two antibiotics are considered largely equivalent. We observed resistance to amoxicillin at a rate of 63.3%, while resistance to amoxicillin–clavulanic acid was 60.8%. Benrabia et al. reported 100% resistance to penicillin and amoxicillin–clavulanic acid in methicillin-resistant *S. aureus* strains found in poultry [58], and Rafiq et al. found a 93.9% resistance rate to ampicillin in their study on livestock in Bangladesh [59]. Penicillins, being one of the oldest antimicrobial classes used in both veterinary and human medicine, typically show high resistance rates, which are consistent with these findings. Although combinations with clavulanic acid are not approved for poultry due to the lack of established residue limits, their inclusion in research provides valuable insights. This is particularly relevant given the frequent production of penicillin-degrading enzymes by *Staphylococcus* strains, which the beta-lactamase inhibitors, such as clavulanic acid, aim to counteract. Resistance is most commonly mediated by the enzymatic cleavage of β-lactam antibiotics, leading to the inactivation of the molecule [60].

For vancomycin, we detected a resistance rate of 18.1%, contrasting with the findings of Benrabia et al. and Argudín et al., who reported no resistance [58,61]. However, our results align with Moawad et al., who documented a resistance rate of 12.8% [62]. Several factors might explain the high resistance levels observed. One contributing factor could be cross-resistance or co-resistance with other antibiotics, as suggested by the strongly positive correlation observed with amoxicillin. Secondly, historical use of the related compound avoparcin as a growth promoter in the poultry industry may have contributed to this resistance. A key aspect of vancomycin’s mechanism of action is its binding to the terminal D-alanyl-D-alanine (D-Ala-D-Ala) motif, a precursor in bacterial cell wall synthesis. In resistant bacteria, this terminal sequence is replaced by D-alanyl-D-lactate (D-Ala-D-Lac), significantly reducing the antibiotic’s binding affinity [63].

Among tetracyclines, a 79.5% resistance rate to doxycycline was observed. Studies by Benrabia et al. reported a higher resistance rate of 96.2% [58], while Argudín et al. and El-Adawy et al. both documented 100% resistance [61,64]. In contrast, Rafiq et al. found a 55.6% resistance rate [59]. The extensive and long-term use of tetracyclines in the poultry industry has likely driven this widespread resistance. Their poor bioavailability [65], often compensated for by overdosing, and the environmental release of unmetabolized active forms have exerted a continuous selective pressure on bacterial populations, exacerbating the issue. Resistance is most commonly driven by the overexpression of efflux pumps and mutations in ribosomal protection proteins [66].

Our study revealed a resistance rate of 68.7% for enrofloxacin compared to 53.2% ciprofloxacin resistance reported by Benrabia et al. [58] and 57.9% enrofloxacin resistance documented by Rafiq et al. [59]. The high resistance rate to this critically important antibiotic, reserved for inpatient care in human medicine, is particularly concerning because of its broad spectrum and high efficiency. Moreover, a portion of enrofloxacin metabolizes into ciprofloxacin, a drug which is extensively used in human healthcare, for example, for multi-resistant urinary tract infections or prostate inflammation. Resistance is often associated with efflux pump activity, and mutations in topoisomerase proteins have also been observed. The emergence of resistance is both rapid and widespread [64].

For potentiated sulfonamides, we observed a resistance rate of 60.2%, while Benrabia et al. reported a lower rate of 28.9% [58], and El-Adawy et al. found all strains to be sensitive [64]. On the other hand, Moawad et al. documented 100% resistance [62], and Rafiq et al. observed a resistance rate of 63.6% [59]. Our findings represent a moderate resistance level, which can be attributed to the long-term and extensive use of this drug class. The significant variability in resistance rates across studies likely reflects differences in antibiotic usage practices among countries, as well as the potential for cross-resistance or co-resistance with other antimicrobials. Resistance most commonly arises from mutations in genes responsible for folic acid synthesis, leading to the production of alternative enzymes involved in biosynthesis [67].

For imipenem, a resistance rate of 12.7% was observed, which aligns closely with the 12.8% resistance rate reported by Moawad et al. [62]. While the comparative literature on imipenem resistance in veterinary isolates is sparse, the fact that this antibiotic is exclusively reserved for critical use in human medicine underscores the undesirability of any resistance. Continuous monitoring of imipenem resistance should be integral to the One Health approach. However, there are stability issues with this compound, as noted in the literature [68], indicating that it remains stable for only a few hours [69] and that increasing temperature accelerates the process [70]; caution is warranted in interpreting these findings. Future studies should consider replacing imipenem with more stable alternatives like meropenem or ertapenem.

For tylosin, we observed a resistance rate of 41.0%, which is consistent with the 43% resistance reported by Lin et al. in poultry samples [71]. In the case of tiamulin, resistance was alarmingly high, at 90.4%, comparable to the 100% resistance rate reported by Nemeghaire et al. [72]. These findings are troubling, given that tiamulin is a broad-spectrum antibiotic widely used in veterinary medicine. In general, three primary mechanisms contribute to resistance: target site modification, efflux pump activity, and enzymatic inactivation [73].

Resistance is also determined by utilization age, farm size, or location, showing notable differences in resistance rates across several antibiotics. This is consistent with our previous studies on chickens, which also showed similar differences based on usage [74,75,76]. These patterns likely reflect age-specific differences in antibiotic usage and the longer rearing period for turkeys, which provides a prolonged window for antibiotic selection pressure on bacteria.

When comparing our findings to human resistance data, resistance rates for macrolides were relatively similar between turkeys (41.0%) and humans (29.9%). For all other antibiotics, resistance levels were markedly higher in veterinary isolates. Fluoroquinolones showed a resistance rate of 68.7% in turkey isolates, compared to just 16.2% in human isolates. Sonola et al. reported 11.7% ciprofloxacin resistance in human strains [77]. The high resistance rates in veterinary isolates highlight the overuse of fluoroquinolones in the poultry industry, underscoring the need for more conscientious stewardship of this drug class to preserve its efficacy for human medicine [78]. For doxycycline, the resistance rate was the highest among the tested antibiotics, at 79.5% in turkey isolates, compared to 5.7% in human isolates. Nazarchuk et al. reported a 34.6% resistance rate in human strains [79]. These findings highlight the critical role of the One Health approach in preserving the effectiveness of life-saving antibiotics for future generations. Achieving this goal requires not only legislative harmonization but also the promotion of responsible antibiotic use and public education to foster a culture of stewardship.

## 4. Materials and Methods

### 4.1. The Origin of Strains and Human Data

The isolates analyzed in this study were collected between February 2022 and May 2023 as part of routine diagnostic sampling conducted by veterinarians serving large-scale poultry farms in collaboration with poultry health experts of the Department of Animal Hygiene, Herd Health and Mobile Clinic of University of Veterinary Medicine, Budapest. Farms were selected randomly for the study, with the primary objective of including at least three farms per region to ensure national coverage. Samples, including 15 oropharyngeal and 15 cloacal swabs per farm, were collected using Amies-type transport medium (Biolab Zrt., Budapest, Hungary). The isolates used for this study were provided by a diagnostic center under a collaborative agreement. Pure cultures were preserved using the Microbank™ system (Pro-Lab Diagnostics, Richmond Hill, ON, Canada) and stored at −80 °C until further analysis. Human resistance data were supplied by the National Public Health and Pharmaceutical Center with approval from the Chief Medical Officer. All participating farms consented to the survey on a voluntary and anonymous basis. Permission was granted to use geographic data at the regional level only.

Samples were categorized by the anatomical source (trachea or cloaca) and assigned by one of Hungary’s seven administrative regions (Supplementary Table S2) to ensure that sample collection was approximately representative. This regional stratification facilitated comparative analysis with human resistance data, which were also provided by the National Public Health and Pharmaceutical Center at regional and national aggregation level.

### 4.2. Minimum Inhibitory Concentration (MIC) Determination

The phenotypic expression of antimicrobial resistance (AMR) was assessed by determining the minimum inhibitory concentration (MIC) values of the bacterial isolates following the guidelines of the Clinical Laboratory Standards Institute (CLSI) [80]. Breakpoints were also determined based on CLSI guidelines [80] and compared with the European Committee on Antimicrobial Susceptibility Testing (EUCAST) epidemiological cutoff values (ECOFFs). For certain antimicrobial agents lacking CLSI-defined breakpoints, such as imipenem [62], tilozin [71], and tiamulin [72], we relied on published data.

Bacterial isolates stored at −80 °C were revived one day before testing by suspending them in 3 mL of cation-adjusted Mueller–Hinton broth (CAMHB, Biolab Zrt., Budapest, Hungary) and incubating at 37 °C for 18–24 h. The tests were conducted using 96-well microtiter plates (VWR International, LLC., Debrecen, Hungary). Except for the first column, all wells were filled with 90 µL of CAMHB. Stock solutions of the tested antimicrobial agents (Merck KGaA, Darmstadt, Germany) at a concentration of 1024 µg/mL were prepared according to CLSI guidelines [80]. From a 512 µg/mL working solution prepared by diluting the stock solution with CAMHB, 180 µL was added to the first column of the microtiter plate, and a two-fold serial dilution was performed. To prepare a two-fold dilution series, 90 µL of liquid was transferred from each well of a column to the corresponding well in the next column. After thorough mixing by pipetting 3–4 times, 90 µL was transferred to the subsequent column, and this process was repeated up to column 10. Excess solution was discarded after the 10th column, leaving 90 µL in each well.

Bacterial suspensions were prepared using a nephelometer (ThermoFisher Scientific, Budapest, Hungary) and adjusted to 0.5 McFarland (10^8^ colony-forming unit (CFU)/mL). These suspensions were inoculated into the microtiter plates from the 11th column backward at a volume of 10 µL per well [79]. Further dilutions, performed according to the CLSI protocol, resulted in all wells containing a final bacterial concentration of 10⁵ CFU/mL. Evaluation was performed using the Sensititre™ SWIN™ automatic MIC reader and VIZION system software version 3.4 (ThermoFisher Scientific, Budapest, Hungary, 2024). *S. aureus* (ATCC 23235) served as the reference isolate. The MIC value is defined as the lowest antibiotic concentration at which no turbidity is observed in the medium, indicating the absence of bacterial growth and the achievement of a bacteriostatic effect.

### 4.3. Statistical Analysis

Data analysis was conducted using R version 4.1.0 [81]. The Shapiro–Wilk test was applied to evaluate the normality of data distribution. For data that deviated from a normal distribution, non-parametric methods were utilized. The Kruskal–Wallis test [82], which does not require normality assumptions, was employed to analyze resistance levels for each active substance across multiple sample groups. This method is particularly effective for comparing medians among groups. To identify specific group differences, post hoc analyses were performed, and pairwise comparisons were carried out using the Mann–Whitney U test [83], and *t*-tests. Bonferroni correction was applied to control for Type I errors due to multiple comparisons [84], although it is acknowledged that this approach can increase the risk of Type II errors (failing to identify true differences). Further analysis included correlation assessments to examine the relationships between individual active substances. Principal component analysis (PCA) [85] was conducted to identify underlying patterns and visualize similarities or differences in the data. Hierarchical cluster analysis followed, and results were depicted in a dendrogram [86], offering a clear representation of isolate clustering and the hierarchical relationships among them.

The essence of correlation analysis lies in examining the strength and direction of the relationship between two or more variables. It reveals how changes in one variable affect the other. This co-movement can be positive (when one variable increases, the other also increases) or negative (when one variable increases, the other decreases). The strength of the relationship is measured by the correlation coefficient, which ranges from −1 to +1. A value of +1 indicates a perfect positive correlation, −1 signifies a perfect negative correlation, and 0 means no correlation between the variables.

Cluster analysis is a statistical method aimed at discovering underlying structures in the data by grouping data points into clusters. These clusters consist of data points that are similar to each other in some respect but different from those in other clusters. A dendrogram is a visual tool used to represent the results of hierarchical cluster analysis. It is a tree-like diagram that illustrates the relationships and hierarchy among the clusters. This approach can be used to infer the distribution of strains exhibiting similar resistance profiles, which may serve as a basis for selecting multidrug-resistant strains for further investigation of genetic determinants using next-generation sequencing.

Principal component analysis (PCA) reduces the dimensionality of data (number of variables) while preserving as much information as possible. This is particularly useful when the dataset contains many variables, making analysis and visualization challenging. PCA creates new variables, called principal components. The first principal component explains the largest variance among the variables, the second principal component explains the second largest variance, and so on. The principal components are independent (orthogonal) of each other, meaning they are uncorrelated.

## 5. Conclusions

Overall, we can conclude that further investigation of putative commensal strains in turkeys is justified based on our results. These strains often serve as natural reservoirs for resistance and play a pivotal role in the dissemination of antimicrobial resistance. Through regular surveillance studies, it is possible not only to provide a situational overview but also to establish temporal trends, identifying patterns in resistance spread. Aligning our findings with human resistance data further reinforces collaborative efforts.

Future studies should focus on incorporating reliable data on antibiotic usage to deepen the understanding of resistance dynamics. Including clinical isolates for comparison would provide a comprehensive perspective, adding a critical third pillar to these investigations. Multi-drug-resistant strains, particularly those resistant to critically important antibiotics, merit detailed analysis through next-generation sequencing to elucidate the genetic mechanisms underlying phenotypic resistance. These efforts underscore the urgency of implementing targeted interventions, such as stricter antibiotic usage protocols and alternative therapeutic strategies, to address high resistance rates. Longitudinal studies linking resistance trends with specific antibiotic usage patterns on farms, combined with advanced genomic approaches, can pave the way for more effective control measures.

## Figures and Tables

**Table 1:** Statistical analysis of resistance based on various criteria.

Antibiotics	Respiratory–Cloaca	Broiler–Breeding	^3^ Young–^4^ Adult	^5^ Small–^6^ Medium
	*p*-Values			
Doxycycline	0.0067 *	0.0067 *	0.0067 *	0.0265 *
Enrofloxacin	0.2669	0.9473	0.9473	0.1141
^1^ Potentiated sulphonamide	0.0097 *	0.1932	0.1932	0.0020 *
Vancomycin	0.3505	0.0137 *	0.0137 *	0.0421 *
Amoxicillin	0.8926	0.0321 *	0.0321 *	0.0729
^2^ Amoxicillin–clavulanic acid	0.5078	0.0043 *	0.0043 *	0.2733
Imipenem	0.3624	0.0157 *	0.0157 *	0.0248 *
Tilozin	0.9904	0.0001 *	0.0001 *	0.0006 *
Tiamulin	0.4776	0.0180 *	0.0180 *	0.2706

Notes: * significant difference (*p* < 0.05); ^1^ trimetoprime–sulphametoxazole, 1:19 ratio; ^2^ 1:2 ratio; ^3^ younger than 13 weeks; ^4^ older than 13 weeks; ^5^ small—5001–50,000; ^6^ medium—50,001–100,000.

**Table 2:** Frequency table of minimum inhibitory concentration (MIC) values for antimicrobial agents with clinical breakpoints in turkey-derived *Staphylococcus* strains. The table presents the MIC values observed for antimicrobial agents with defined clinical breakpoints in *Staphylococcus* isolates from turkeys (*n* = 166). For each antimicrobial agent, the upper row displays the number of isolates, while the lower row shows the corresponding percentage. Vertical red lines indicate the clinical breakpoints for each agent. The MIC_90_ of the study population exceeded the clinical breakpoint for all antibiotics, indicating that at least 90% of the population was resistant. However, when assessing MIC_50_ (the concentration at which 50% of the population is inhibited), amoxicillin–clavulanic acid, imipenem, vancomycin, and tilozin remained below the clinical breakpoint.

Antibiotics	^1^ BP *	0.001	0.002	0.004	0.008	0.016	0.03	0.06	0.125	0.25	0.5	1	2	4	8	16	32	64	128	256	512	1024	MIC_50_	MIC_90_	^2^ ECOFF
	µg/mL																						µg/mL		
Amoxicillin	0.5				1	0	4	11	22	23	13	11	15	14	16	3	12	0	1	6	11	3	1	256	0.5
					0.6%	0.0%	2.4%	6.6%	13.3%	13.9%	7.8%	6.6%	9.0%	8.4%	9.6%	1.8%	7.2%	0.0%	0.6%	3.6%	6.6%	1.8%			
Doxycycline	0.5	1	1	2	6	3	1	3	11	6	10	28	18	21	13	21	10	11					2	32	0.5
		0.6%	0.6%	1.2%	3.6%	1.8%	0.6%	1.8%	6.6%	3.6%	6.0%	16.9%	10.8%	12.7%	7.8%	12.7%	6.0%	6.6%							
^3^ Amoxicillin-clavulanic acid	1				1	0	3	11	18	16	16	17	19	19	22	17	6	1					0.25	16	0.5
					0.6%	0.0%	1.8%	6.6%	10.8%	9.6%	9.6%	10.2%	11.4%	11.4%	13.3%	10.2%	3.6%	0.6%							
Tiamulin	4									2	3	5	6	2	3	13	22	35	29	18	14	14	64	512	2
										1.2%	1.8%	3.0%	3.6%	1.2%	1.8%	7.8%	13.3%	21.1%	17.5%	10.8%	8.4%	8.4%			
Enrofloxacin	4					3	1	8	6	7	10	6	11	20	8	18	21	35	7	5			16	64	0.5
						1.8%	0.6%	4.8%	3.6%	4.2%	6.0%	3.6%	6.6%	12.0%	4.8%	10.8%	12.7%	21.1%	4.2%	3.0%					
^4^ Potentiated sulphonamide	4												8	7	19	14	18	20	23	8	12	37	4	64	0.25
													4.8%	4.2%	11.4%	8.4%	10.8%	12.0%	13.9%	4.8%	7.2%	22.3%			
Imipenem	8	1	0	0	3	10	24	20	10	21	17	16	9	14	13	6	2						0.063	8	0.125
		0.6%	0.0%	0.0%	1.8%	6.0%	14.5%	12.0%	6.0%	12.7%	10.2%	9.6%	5.4%	8.4%	7.8%	3.6%	1.2%								
Vancomycin	32							1	16	37	26	38	8	3	3	4	5	0	0	18	7		1	256	2
								0.6%	9.6%	22.3%	15.7%	22.9%	4.8%	1.8%	1.8%	2.4%	3.0%	0.0%	0.0%	10.8%	4.2%				
Tilozin	64					1	2	1	8	11	13	35	9	10	3	4	1	1	6	6	16	39	4	1024	2
						0.6%	1.2%	0.6%	4.8%	6.6%	7.8%	21.1%	5.4%	6.0%	1.8%	2.4%	0.6%	0.6%	3.6%	3.6%	9.6%	23.5%			

* BP—breakpoint; ^1^ Clinical Laboratory Standard Institute (CLSI); ^2^ Epidemiological cut-off value (EUCAST); ^3^ 2:1 ratio; ^4^ trimetophrime–sulphamethoxazole, 1:19 ratio.

## Data Availability

The data presented in this study are available from the corresponding author upon reasonable request.

## Supplementary Materials

The following supporting information can be downloaded at: https://www.mdpi.com/article/10.3390/antibiotics14020200/s1. Table S1: Frequency table of the minimum inhibitory concentration (MIC) values (µg/mL) for agents without breakpoints in *Staphylococcus* samples derived from turkeys (*n* = 166). Table S2: The seven administrative regions of Hungary in the original language and their English translations.
