# Supplementary material for: Antimicrobial Susceptibility Profiles of Commensal Staphylococcus spp. Isolates from Turkeys in Hungarian Poultry Farms Between 2022 and 2023
Source: Antibiotics (Basel). 2025 Feb 14;14(2):200. doi: 10.3390/antibiotics14020200 (PMC11851855; doi:10.3390/antibiotics14020200)
Supplement: Supplementary file 1 [file antibiotics-14-00200-s001.zip › antibiotics-3457128-supplementary.pdf]

Supplementary Table S1 Frequency table of the minimum inhibitory concentration (MIC) values (µg/mL) for agents without breakpoints in *Staphylococcus* samples derived from turkeys (*n*=166). The top row for each agent shows the count, while the bottom row shows the percentage.

Antibiotic	0.001	0.002	0.004	0.008	0.016	0.03	0.06	0.125	0.25	0.5	1	2	4	8	16	32	64	128	256	512	1024	MIC_{50}	MIC_{90}	[1]ECOFF
																						µg/mL		
**Ceftriaxone**							3	2	5	18	21	19	19	21	25	6	4	2	10	8	3	4	256	-
							1.8%	1.2%	3.0%	10.8%	12.7%	11.4%	11.4%	12.7%	15.1%	3.6%	2.4%	1.2%	6.0%	4.8%	1.8%			
**Neomycin**								2	4	8	31	8	9	25	34	14	12	6	0	12	1	8	128	-
								1.2%	2.4%	4.8%	18.7%	4.8%	5.4%	15.1%	20.5%	8.4%	7.2%	3.6%	0.0%	7.2%	0.6%			
**Spectinomycin**														1	4	32	42	55	21	2	9	128	256	128
														0.6%	2.4%	19.3%	25.3%	33.1%	12.7%	1.2%	5.4%			
**Florfenicol**										6	3	27	53	43	16	3	8	6	1			4	32	8
										3.6%	1.8%	16.3%	31.9%	25.9%	9.6%	1.8%	4.8%	3.6%	0.6%					
**Lincomycin**											2	9	11	25	32	21	10	9	5	26	16	32	512	2
											1.2%	5.4%	6.6%	15.1%	19.3%	12.7%	6.0%	5.4%	3.0%	15.7%	9.6%			
**Colistin**														25	7	19	10	20	14	39	32	256	1024	-
														15.1%	4.2%	11.4%	6.0%	12.0%	8.4%	23.5%	19.3%			

[1]epidemiological cut-off value (EUCAST)

Supplementary Table S2 The seven administrative regions of Hungary in the original language and their English translations

Hungarian name	English name
Dél-Alföld	Southern Great Plain
Közép-Dunántúl	Central Transdanubia
Közép-Magyarország	Central Hungary
Észak-Magyarország	North Hungary
Nyugat-Dunántúl	West Transdanubia
Dél-Dunántúl	South Transdanubia
Észak-Alföld	Northern Great Plain
